# Metastatic Breast Cancer Presenting as Acute Appendicitis

**DOI:** 10.7759/cureus.30456

**Published:** 2022-10-19

**Authors:** David T Khalil, Kellee Slater, Caroline Cooper

**Affiliations:** 1 Medicine, Queensland Health, Brisbane, AUS; 2 Surgery, University of Queensland, Brisbane, AUS; 3 Anatomical Pathology, Princess Alexandra Hospital, Brisbane, AUS

**Keywords:** acute appendicitis, cancer survival, general surgery and breast cancer, rare cause of acute abdominal pain, breast cancer metastasis

## Abstract

Breast cancer is one of the most commonly diagnosed cancers in Australia. With the development in screening, diagnosis, and treatment, people are living longer with metastatic disease of the breast. This malignancy commonly metastasizes to the lung, brain, bone, and liver. However, due to the increased survival of patients living with breast cancer, metastases may present with complications that have not been seen before. We describe a case of a 55-year-old female with a background of metastatic breast cancer to the brain who presented to the emergency department with acute appendicitis. At surgery, a malignant mass was found in the cecum obstructing the appendix, and histopathology revealed metastatic breast cancer.

## Introduction

Breast cancer is the third most frequently diagnosed cancer in Australia, surpassed only by skin and prostate cancer [1]. In 2021, breast cancer accounted for approximately 13% of all new cancers diagnosed [2]. Mortality rates of patients diagnosed with breast cancer continue to improve, with a five-year survival of 92% recorded for 2013-2017 compared to 76% in 1988-1992 [3]. This high survival is being driven by national screening programs resulting in early detection and ever-evolving surgical, radiological, and systemic treatment strategies that are utilized [4]. This long-term disease survival raises the possibility of unexpected metastatic sites, and there is some suggestion that sites of breast cancer metastasis have changed in distribution [5].

The most common sites for breast cancer metastases are the bone, lung, brain, and liver [6]. Similar to other types of metastatic malignancy such as melanoma, patients living with breast cancer have the potential to present with an acute surgical abdomen caused by malignant foci. In patients with a known history of advanced breast cancer, the metastatic disease should form part of the differential diagnosis as a cause for an acute abdomen, as demonstrated in this case [7]. We present a case of a metastatic breast cancer to the base of the appendix presenting as acute appendicitis.

## Case presentation

A 55-year-old female attended the emergency department with a two-day history of colicky periumbilical abdominal pain. The pain then became localized to the right iliac fossa and was aggravated by movement and coughing. She reported mild anorexia and one episode of loose stool the day prior but had not passed any flatus or feces since. Furthermore, she denied any recent nausea, vomiting, or infective or gynecological symptoms.

Her past medical history was significant for metastatic breast cancer first diagnosed six years prior to this presentation. The histology of her cancer when first diagnosed revealed an estrogen receptor (ER)-positive, progesterone receptor (PR)-positive, and human epidermal growth factor receptor 2 (HER-2)-negative grade III invasive ductal carcinoma (T4 N2) with lymphovascular invasion. This was managed with a modified radical mastectomy of the right breast, followed by adjuvant chemotherapy, radiotherapy to the chest wall, and hormonal therapy with anastrozole. Four years later, she was diagnosed with several brain metastases while on hormonal therapy for which she received a combination of surgical debulking and Gamma Knife radiotherapy. Her hormonal therapy was changed to exemestane, which she continued to take at the time of presentation.

Because of these treatments, despite her metastatic disease, her quality of life was excellent, and recent imaging of the treated brain disease showed no progression. Apart from her metastatic breast cancer, she had no other comorbidities, was not a smoker, and was working in manufacturing. Her surgical history included two caesarean sections, and she had not had a recent colonoscopy.

Examination on presentation revealed a heart rate of 106 beats per minute and a temperature of 37.6 degrees Celsius. Her abdominal examination revealed maximal tenderness in the right iliac fossa with signs of peritonism. Rovsing’s sign was positive. There was no palpable mass.

**Figure 1:**
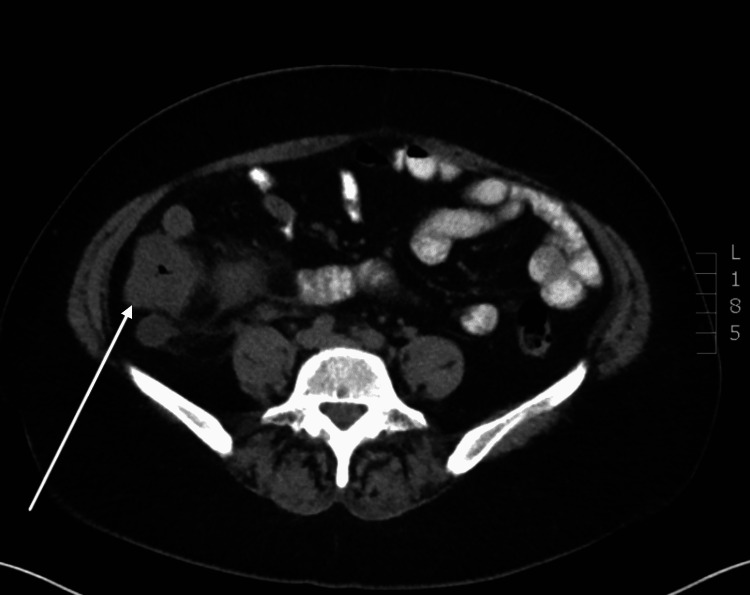
CT scan of the abdomen and pelvis with oral contrast (axial slice). Arrow indicating cecal thickening just posterior the engorged appendix. CT: computed tomography

Full blood count, electrolyte, renal function, and liver function tests were performed, revealing only mild elevation of the white cell count to 13.4 × 109/L (4-11). Urinary microscopy was negative for white cells and bacteria. Computed tomography (CT) scan, performed with oral contrast due to previous reaction to intravenous contrast, revealed acute appendicitis with severe dilatation of the proximal appendix up to 20 mm (Figure 1).

**Figure 2:**
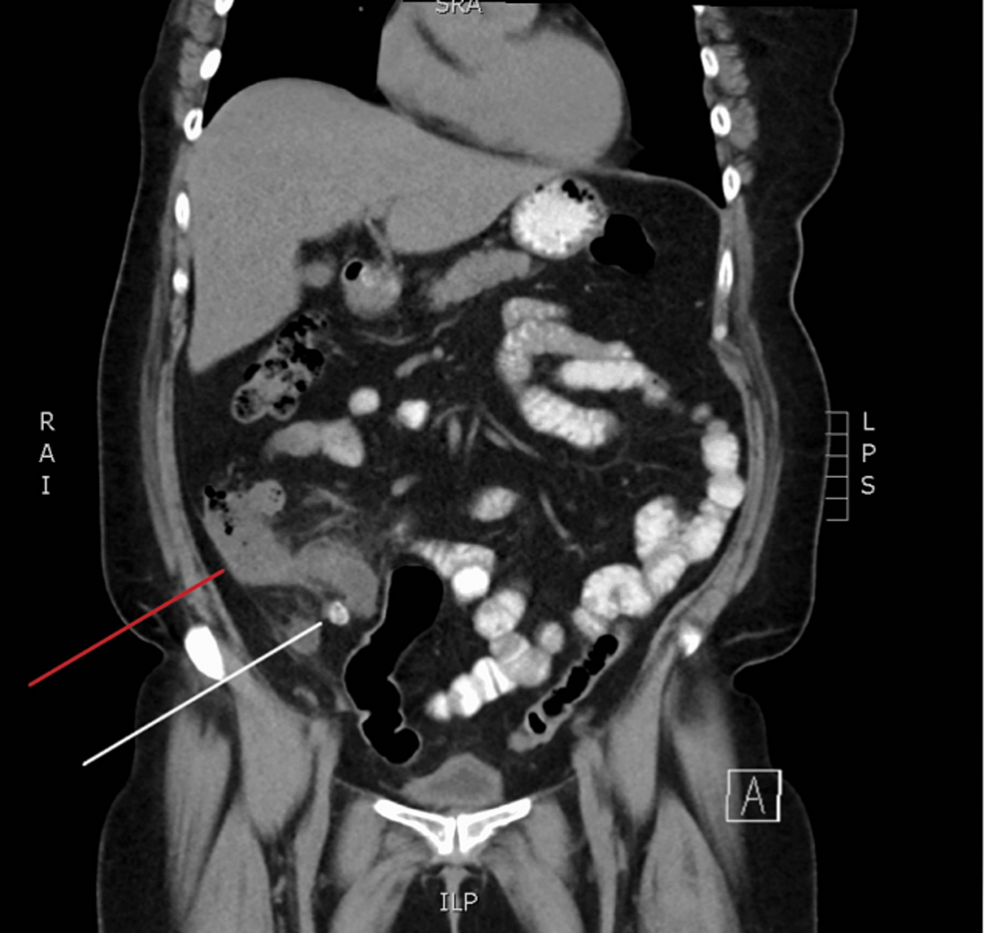
CT scan of the abdomen and pelvis with oral contrast (coronal slice). Red marker indicating cecal thickening. White marker indicating fecalith. CT: computed tomography

The mesoappendix was markedly engorged, and the posterior margin was poorly defined, raising suspicion of devitalization and imminent perforation. There was moderate adjacent-free fluid and a circumscribed, calcified area that could have represented a fecalith. The cecum was bulky. There were no signs of bowel obstruction (Figure 2).

Clinically, the patient’s presentation was suggestive of appendicitis; however, given her age and the long history of metastatic malignancy and the abnormal appearance of the cecum, there was a high index of suspicion that the appendicitis may be the result of an obstructing, malignant cecal pathology. Surgical management was discussed with the patient including the possibility that this may be more complicated than simple appendicitis. In addition to appendicectomy, she agreed to undergo colectomy and/or ileostomy if required.

Intra-operative findings were of suppurative inflammation with the appendix perforated at its base. A fecalith was identified sitting free in an abscess cavity surrounded by 20 mL of contained pus. The cecum was severely deformed with inflammation, and it was difficult to assess laparoscopically whether a mass in the cecum was present. Given the index of suspicion for an obstructing lesion at the base of the appendix, the right colon was mobilized laparoscopically and then delivered via a midline incision. A cecectomy was performed using a stapling device, maintaining the patency of the ileocecal valve. The specimen was opened on the back table to ensure a more extensive colectomy was not required. There was an ulcerated lesion present at the appendix orifice, but macroscopically, it appeared clear of the resection margin. The specimens were sent for histology. There was no evidence of lymphadenopathy or other peritoneal disease suggesting widespread peritoneal malignancy.

**Figure 3:**
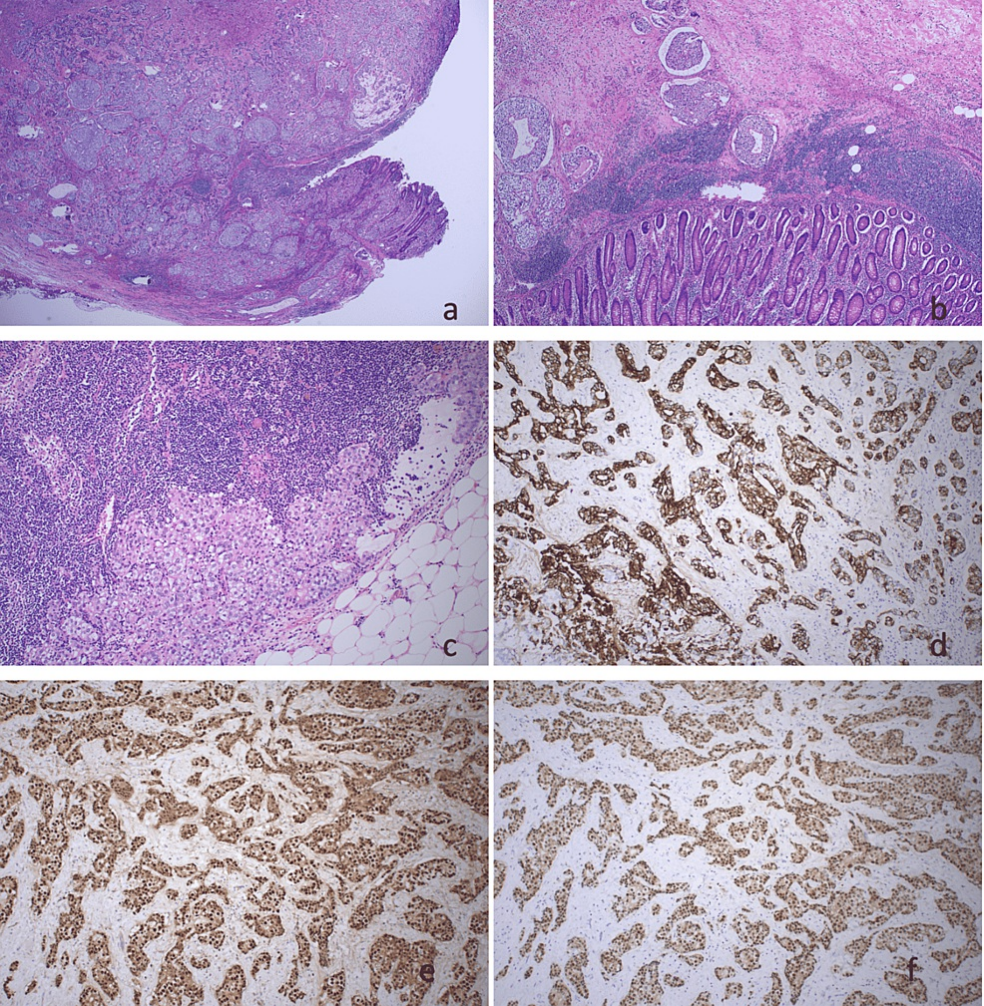
Histology and immunohistochemistry of excised tumor. (a) Low power of the tumor causing the ulceration of the appendix. The tumor is in cohesive nests. There is ulceration of the mucosa, and the tumor involved the serosal surface where there was perforation. (b) Medium power of the tumor in lymphovascular spaces in the submucosa. (c) Medium power of lymph node metastasis. (d) Immunohistochemistry of CK7. (e) Immunohistochemistry of ER. (f) Immunohistochemistry of GATA3 CK7: cytokeratin 7; ER: estrogen receptor

Recovery was uncomplicated, and after a course of intravenous antibiotics, the patient was discharged on post-operative day 4. The histology of the resected specimen was consistent with ER-positive, PR-negative, HER-2-negative metastatic breast carcinoma. An 18 mm tumor was identified in the proximal appendix with evidence of lymphovascular invasion and extension onto the visceral peritoneum. Microscopic surgical margins were clear (Figure 3).

The case was discussed at the general surgery multidisciplinary meeting, and the consensus was that no further surgical intervention was required. The patient was returned to her oncologist for ongoing management where she continued on her hormonal therapy.

Three months following the appendicectomy, the patient developed bilateral ureteric obstruction secondary to peritoneal carcinomatosis and required bilateral nephrostomy tubes and ureteric stenting. She has undergone further chemotherapy that has controlled her peritoneal disease enough to allow the removal of the ureteric stents and nephrostomy tubes. She has most recently developed a liver metastasis that is being treated with radiotherapy. Her quality of life remains excellent seven years after the initial diagnosis of breast cancer, with minimal symptoms from her metastases, and she is independent with all her activities of daily living.

## Discussion

Appendicitis secondary to breast cancer metastases is not common; however, there are a number of cases documented in the published literature. In 1998, Connor et al. performed a retrospective analysis of histopathology results from appendicectomies and highlighted the rarity of cancer metastases in the appendix. Of the 7,970 results analyzed, 0.9% were identified to contain tumors. Carcinoid tumors were the most common. Only 15% of all tumors identified were secondary malignancies, the majority being metastatic colorectal cancer [7]. A similar study was conducted on a smaller scale by Esmer-Sánchez et al. published in 2004, and these researchers came to similar conclusions [8]. A literature review, presented in Table 1, highlights recent documented cases of metastatic breast cancer presenting as acute appendicitis (within the last 20 years).

**Table 1:** Literature review documenting cases of metastatic breast cancer presenting with acute appendicitis. ER: estrogen receptor; PR: progesterone receptor; HER-2: human epidermal growth factor receptor 2; RM: radical mastectomy; F: female

Publishing author	Year published	Patient details	Breast cancer type (receptor status)	Treatment
Pate et al. [9]	2021	59, F	Invasive ductal carcinoma (receptor-negative)	Appendicectomy and neoadjuvant chemotherapy prior to RM
De Pauw et al. [10]	2020	64, F	Invasive ductal carcinoma (ER-positive)	Appendicectomy and refused further therapy
Amigo et al. [11]	2000	49, F	Invasive ductal carcinoma (not specified)	Appendicectomy
Numan et al. [12]	2019	44, F	Invasive lobular carcinoma (ER-positive, PR-positive, and HER-2-negative)	Ileocecectomy and appendicectomy
Ng et al. [13]	2018	59, F	Invasive ductal carcinoma (ER-negative, PR-positive, and HER-2-positive)	Right hemicolectomy, followed by RM, adjuvant chemotherapy, and radiotherapy
Araujo et al. [14]	2018	37, F	Invasive ductal carcinoma (ER-negative, PR-negative, and HER-2-positive)	Appendicectomy, hysterectomy and left oophorectomy, and neoadjuvant chemotherapy prior to RM
Kwan et al. [15]	2016	70, F	Invasive ductal carcinoma (ER-positive and PR-positive)	Continued hormonal therapy
Mori et al. [16]	2016	56, F	Invasive ductal carcinoma (ER-positive, PR-positive, and HER-2-positive)	Appendicectomy
Dirksen et al. [17]	2010	76, F	Invasive lobular carcinoma (ER-positive, PR-negative, and HER-2-negative)	Appendicectomy
Pigolkin et al. [18]	2008	60, F	Cancer type not specified (ER-positive and PR-positive)	Appendicectomy
Varga et al. [19]	2005	45, F	Invasive ductal carcinoma (not specified)	Appendicectomy, followed by RM

In an extensive review of rare sites of breast cancer metastases conducted by Di Micco et al. (2019), they found that metastatic disease in less common sites is increasing and frequently developed in conjunction with metastases to other more common sites. It was unusual to find an isolated metastasis to a rare site, without it being preceded by a metastasis to a well-established target organ such as the brain, lung, bone, and liver. Metastases to the gastrointestinal (GI) tract as a whole were found to be rare, and according to their review, chemotherapy and hormonal therapy were often used, but early detection and surgical excision when possible proved vital. They concluded that the increased incidence of unusual sites of breast cancer metastases is secondary to prolonged survival of patients with metastatic breast cancer but may also represent the progression of disease, which is resistant to conventional therapies [20].

## Conclusions

Due to the availability of multiple modalities that provide local disease control, as well as breast cancer hormone, targeted, and systemic treatments, patients are surviving longer with metastatic disease. We have described a case of a patient who was living with breast cancer metastases to the brain and peritoneum.

When patients with a long history of metastatic disease present with abdominal signs and symptoms, complications related to malignancy must be high in the differential diagnosis. As the limited research on the topic indicates, early detection and surgical intervention are keys in determining outcomes for such patients. Not only is this true in regard to the progression of their metastatic disease, but also early intervention can be lifesaving by preventing severe complications such as sepsis, as evidenced in the above case of perforated appendicitis. Finally, it is vital for clinicians to have this high index of suspicion so that informed discussion can take place prior to surgery to identify the patient’s wishes with regard to quality of life and palliative procedures.
